# Nanocarriers From Natural Lipids With *In Vitro* Activity Against *Campylobacter jejuni*

**DOI:** 10.3389/fcimb.2020.571040

**Published:** 2021-01-08

**Authors:** Lígia Nunes de Morais Ribeiro, Eneida de Paula, Daise Aparecida Rossi, Flávia Alves Martins, Roberta Torres de Melo, Guilherme Paz Monteiro, Márcia Cristina Breitkreitz, Luiz Ricardo Goulart, Belchiolina Beatriz Fonseca

**Affiliations:** ^1^ School of Veterinary Medicine, Federal University of Uberlandia, Uberlândia, Brazil; ^2^ Institute of Biotechnology, Federal University of Uberlandia, Uberlândia, Brazil; ^3^ Department of Biochemistry and Tissue Biology, Institute of Biology, University of Campinas, Campinas, Brazil; ^4^ Department of Analytical Chemistry, Institute of Chemistry, University of Campinas, Campinas, Brazil

**Keywords:** lipid nanoparticles, Campylobacteriose, natural oils, bioactive molecules, biofilm

## Abstract

*Campylobacter jejuni* (CJ) is the most prevalent zoonotic pathogen of chicken meat and related products, which may lead to gastroenteritis and autoimmune diseases in humans. Although controlling this bacterium is important, CJ strains resistance against traditional antibiotic therapy has been increased. Vegetable oils and fats are natural biomaterials explored since the Ancient times, due to their therapeutic properties. Nanotechnology has promoted the miniaturization of materials, improving bioavailability and efficacy, while reducing the toxicity of loaded active molecules. In this work, a screening of 28 vegetable oils was firstly performed, in order to select anti-CJ candidates by the disc diffusion test. Thus, the selected liquid lipids were used as active molecules in nanostructured lipid carriers (NLC) formulations. The three resultant systems were characterized in terms of particle size (~200 nm), polydispersity index (~0.15), and zeta potential (~-35mV), and its physicochemical stability was confirmed for a year, at 25°C. The structural properties of NLC were assessed by infrared (FTIR-ATR) and differential scanning calorimetry (DSC) analyses. The spherical nanoparticle morphology and narrow size distribution was observed by transmission electron microscopy (TEM) and field emission scanning electron (FE-SEM) analyses, respectively. Then, the *in vitro* antimicrobial activity test determined the minimum inhibitory concentration (MIC) of each formulation against CJ strains, in both free (1–3 mg/ml^−1^) and sessile (0.78 mg/ml^−1^) forms. Finally, the *in vitro* biocompatibility of NLC was demonstrated through cell viability using VERO cell line, in which F6 was found twice less cytotoxic than pure olibanum oil. Considering the abovementioned achieved, F6 formulation is able to be evaluated in the *in vivo* anti-CJ efficacy assays.

## Introduction

*Campylobacter jejuni* (CJ) is a Gram-negative commensal pathogen prevalent in the intestinal tract of chickens and other animals. Human infection is characterized by inflammatory bowel response, followed by bloody diarrhea and painful symptoms to the affected people and pets. In addition, Guillain-Barre syndrome can be a secondary sequelae, characterized by a potential fatal autoimmune disorder in humans (Crofts et al., 2018). Moreover, CJ multidrug resistance to different antibiotics used in poultry and humans has been currently noticed. It was described that children and pets with CJ infections were resistant to ceftiofur, sulphazotrim, norfloxacin, tetracycline, amoxicillin, ciprofloxacin, cefazolin, and erythromycin antibiotics (Rodrigues et al., 2015). Furthermore, another work showed that CJ isolated from chicken carcasses were highly resistant to norfloxacin, erythromycin, and amoxicillin, the most used drugs in traditional antibiotic therapy (Fonseca et al., 2016).

Natural oils and fats are employed as drugs in traditional medicine since the Antiquity. Plants synthesize more than 8,000 phytochemicals as defense against pathogens (Klančnik et al., 2020). Different classes of molecules, such as: triglycerides, fatty acids, and small fractions of natural antioxidants (Attama et al., 2006), exert several therapeutic effects, as analgesic, antimicrobial, anti-inflammatory, antifungal, and antineoplastic (Orhan et al., 2010). The antimicrobial and antibiofilm activities of different natural lipids against CJ have been reported, as observed for oregano and cinnamon essential oils (Clemente et al., 2020; Yu et al., 2020). However, despite these combined therapeutic properties, the clinical use of vegetable lipids—mainly the essential oils—are prevented, due to its physicochemical instability, insolubility, high toxicity, volatility, and photosensitivity (Odeh et al., 2014).

In this sense, pharmaceutical nanotechnology provides nanostructured DDS with huge superficial area to specifically interact with the targets (de Araújo et al., 2019). Such systems have moved attention in the last years, due to its impressive properties, such as: the improvement of drug bioavailability, half-time, stability, and efficacy, without affecting the biocompatibility (Ribeiro et al., 2019). Nanostructured lipid carrier (NLC) is a biocompatible DDS composed of an internal lipid matrix (blend of solid and liquid lipids) stabilized by at least one surfactant (Muller et al., 2011). This multi-faceted system is designed to load hydrophobic molecules with efficiency, exhibiting long-term stability at room temperature and scaling up feasibility (Rodrigues da Silva et al., 2020). Different classes of drugs have been loaded for several applications (Souto et al., 2020). In this sense, the encapsulation of natural oils by NLC can be a versatile strategy to preserve its therapeutics properties, prevent degradation, improve the stability, and minimize the toxicity. In fact, there are some works that described the encapsulation of different natural oils as active molecules of NLC with anti-inflammatory (Carbone et al., 2018), antifungal (Badea et al., 2015a), UV protection (Badea et al., 2015b), antineoplastic (Venturini et al., 2015), and bactericidal properties (Khezri et al., 2020). Although, there is still no report of vegetable oils loaded NLC with anti-CJ activity, until now.

This work describes the use of bioactive vegetable oils against CJ, employing nanotechnology. A screening of 28 natural oils with potential anti-CJ activity was performed to select the most desirable liquid lipids for subsequent NLC preparation. The resultant formulations were followed by the long-term stability study (25°C) and their molecular structure was clarified. *In vitro* antimicrobial activity and viability tests were used to select the best NLC formulation regarding anti-CJ and biocompatibility properties. It was also provided detailed structural information of this promisor candidate to be tested in further *in vivo* efficacy tests.

## Materials and Methods

### Screening of Vegetable Oils

Different vegetable and essentials oils (Engenharia das Essências^®^), totalizing 28 samples (**Table 1**), were preliminarily evaluated regarding the anti-CJ activity in disc diffusion test (the description of the experiment was detailed in *in vitro* antimicrobial tests). The samples that exhibited some anti-CJ activity (**Table 2**) were selected to be used as liquid lipid excipients in NLC preparation (**Table 3**).

**Table 1:** Vegetable oils tested against *Campylobacter jejuni* (CJ) by the disc diffusion test.

Sample	Oil
O1	Pequi
O2	Lavender
O3	Garlic
O4	Moringa
O5	Copaiba
**O6**	**Olibanum EO**
O7	Black pepper EO
O8	Basil EO
O9	Lucuma EO
O10	Ginger EO
O11	Sesame
O12	Rosemary EO
O13	Peppermint EO
**O14**	**Salvia EO**
O15	Chia
O16	Patauá
O17	Obliphica
O18	Linseed
**O19**	**Candeia**
O20	Tamanu
O21	Anise
O22	Jojoba
O23	Cacay
O24	Green coffee
O25	Grape seed
O26	Passion fruit
O27	Pracaxi
O28	Mandarin

The samples in bold showed anti-CJ activity, given by the growth inhibition diameter (see **Table 2**). EO, essential oil.

**Table 2:** Results of antimicrobial *in vitro* activity of pure oils (O6, O14, and O19) and NLC (F6, F14, and F19), in terms of the diameter of growth inhibition zone (IZ) and the minimum inhibitory concentration (MIC) against CJ strains, in free and sessile forms (n = 3).

Sample	free form IZ _IAL 2383_(mm)	free form MIC_IAL 2383_(mg.ml^−1^)	free form MIC_10_(mg.ml^−1^)	sessile form MIC_IAL 2383_(mg.ml^−1^)	sessile form MIC_IAL 468_(mg.ml^−1^)	sessile form MIC_IAL 520_(mg.ml^−1^)
O6	21.00 ± 1.40	–	–	–	–	–
F6	36.00 ± 5.70^*^	1.56 ± 0.00	2.60 ± 0.90	0.78 ± 0.00	0.78 ± 0.00	0.78 ± 0.00
O14	23.00 ± 7.00	–	–	–	–	–
F14	35.00 ± 2.80^*^	1.56 ± 0.00	1.56 ± 0.00	NIB	NIB	NIB
O19	28.00 ± 2.80	–	–	–	–	–
F19	43.00 ± 1.40^*^	1.25 ± 0.00	1.56 ± 0.90	NIB	NIB	NIB

Unpaired t-test was used to calculate statistically significant differences of IZ (mm) values between NLC and its respective controls (pure oils); ^*^p < 0.05.

O6 (olibanum essential oil), O14 (salvia essential oil), and O19 (candeia oil) were the liquid lipids used as bioactive molecules in F6, F14, and F19 formulations, respectively. NIB, no inhibition of CJ in biofilms.

**Table 3:** Solid and liquid lipids employed as matrices of nanostructured lipid carrier (NLC) formulations.

Formulation	Solid Lipid	Liquid Lipid
F6	Ucuuba butter (100 mg/ml)	Olibanum EO (50 mg/ml)
F14	Shea butter (100 mg/ml)	Salvia EO (50 mg/ml)
F19	Shea butter (60 mg/ml)	Candeia oil (40 mg/ml)

The formulations were composed of Plantaren^®^ (20 mg/ml) as surfactant and Pluronic 188^®^ (10 mg/ml) as co-surfactant.

### Nanostructured Lipid Carrier Preparation Method

NLC formulations composed of different lipid matrices (**Table 3**) were prepared by the emulsification-ultrasonication method (Ribeiro et al., 2016). Briefly, the solid and liquid lipids were heated in a water bath (10°C above the melting point of solid lipid). Simultaneously, an aqueous solution of Plantaren^®^ plus Pluronic 188^®^ was heated to the same temperature of the lipid phase and added to the oily phase, under high-speed agitation (10,000 rpm), for 2 min with an Ultra-Turrax blender (IKA WerkeStaufen, Germany). Then, the systems were ultrasonicated for 12 min in a Vibracell tip sonicator (Sonics & Mat. Inc., Danbury, USA) operated at 500 W and 20 kHz, in alternating 30 s (on/off) cycles. Finally, the resultant formulations were cooled to room temperature. The resultant NLC and the concentration of each excipient used were displayed below (**Table 3**).

### Physicochemical Stability Study

The physicochemical stability of F6, F14, and F19 formulations was monitored for a year (25°C). The parameters analyzed were nanoparticle size (nm), polydispersity index (PDI), and zeta potential (mV), evaluated by dynamic light scattering (DLS) using in a ZetaSizer ZS90 (Malvern Instruments, UK). The measurements were performed in triplicate (25°C) and ANOVA/Tukey tests were employed (p < 0.05) for statistical analysis, calculated by R software.

### *In Vitro* Antimicrobial Activity Tests

The *in vitro* antimicrobial activity tests were performed as follows: i) preliminarily disc diffusion test using IAL 2383 CJ strain; ii) determination of minimum inhibitory concentration (MIC) from free forms, through IAL 2383 and 10 (isolated from chicken meat) CJ strains; iii) determination of MIC from sessile forms organized in biofilms, using IAL 2383, IAL 468, and IAL 520 (isolated from chicken meat) CJ strains.

Briefly, all the CJ strains were inoculated in CCDA-Preston agar (Oxoid^®^) separately, being incubated in microaerophilic for 48 h. Typical colonies were collected and diluted in 10 ml of sterile saline solution (0.9%), adjusted according 0.5 McFarland scale, and confirmed by the plate count (1.5 × 10^8^ CFU ml^−1^ of final concentration). Each bacterial suspension was then diluted in 96-well plate wells reaching the final concentration of 1 × 10^5^ CFU·ml^−1^ per well (Melo et al., 2019). The resultant bacterial suspensions were used in the disc diffusion and MIC determination tests.

#### Disc Diffusion Test

Disc diffusion test evaluated IAL 2383 CJ strain susceptibility to 28 vegetable oils (screening) before NLC preparation. Then, NLC were prepared and F6, F14, and F19 formulations were also tested in here. The experiment was carried out according to the standard M2-A8 from Clinical Laboratory Standard Institute (CLSI, 2020). Briefly, 20 μl of each pure oil (**Table 1**), F6, F14, and F19 formulations and 0.9% NaCl (control), totalizing 32 samples, were added in sterile filter discs (6 mm of diameter) and stored by 30 min, for drying samples (25°C). Simultaneously, the plates that were previously prepared with Mueller-Hinton agar plus 5% lysed sheep blood (Laborclin^®^), were inoculated with CJ IAL 2383 suspensions adjusted to 0.5 McFarland, as already described. After 10 min, the impregnated discs were positioned over the agar. The plates were incubated in microaerophilic at 37°C for 48 h. After this period, the diameters of CJ growth inhibition zone (mm) was measured for each sample (n = 2) (Duarte et al., 2016).

#### Minimum Inhibitory Concentration Determination in *Campylobacter jejuni* Free Forms

The minimum inhibitory concentration (MIC) of F6, F14, and F19 formulations were determined against free forms of IAL 2383 and 10 CJ strains, through microdilution method (Duarte et al., 2016). IAL 2383 and 10 CJ suspensions were prepared and adjusted to 0.5 McFarland. They were diluted in Muller Hinton Broth (Oxoid^®^), supplemented with cation-adjusted (20–25 mg/L Ca^2+^, 10–12.5 mg/L Mg^2+^) solution plus 5% lysed sheep blood (Laborclin^®^), in accordance with ISO 20776-121 (EUCAST, 2020). Then, different concentrations of F6, F14, and F19 formulations were added in 96-well plates to a final volume of 0.1 ml. The negative control was prepared with Muller Hinton Broth without bacteria (Oxoid^®^), supplemented with cation-adjusted solution plus 5% lysed sheep blood (Laborclin^®^), as detailed above. Finally, the plates were incubated at 37°C, for 48 h in conditions of microaerophilia. After this period, the MIC values was determined based on the lowest concentration of formulation that prevented the bacterial growth. The minimum inhibitory concentration (MIC) was obtained for each formulation (n = 3). ANOVA/Tukey tests were the statistical methods adopted, determined by R software (p < 0.05).

#### Minimum Inhibitory Concentration in Sessile Forms (Biofilms)

The formation of biofilms was carried out according Sulaeman and co-workers (Sulaeman et al., 2010), with modifications. IAL 2383 (Fonseca et al., 2014), 468, and 520 (isolated from chicken meat) CJ strains were used. Concisely, the strains were grown on CCDA agar (Oxoid^®^) and transferred to 20 ml of Mueller Hinton broth (Difco^®^) supplemented with 5% chicken juice, being incubated at 37°C for 48 h under microaerophilic condition. Then, the bacterial suspension was standardized to an OD600 = 0.22 to 0.28 absorbance and centrifuged at 5,000 rpm for 10 min. After supernatant removal, the cells were washed twice, and the pellet was re-suspended in 0.9% NaCl solution. Thus, it was diluted in 10 ml of supplemented MH broth with 5% of chicken juice, obtaining a final count of 10^4^ CFU/ml. CJ suspension (200 µl) was added to 96-well plates and incubated for 48 h at 37°C, under microaerophilic conditions. Afterwards, the non-adherent bacteria were washed twice with 0.9% sterile NaCl solution.

Different concentrations of F6, F14, and F19 formulations were tested for the determination of CJ antimicrobial susceptibility in the sessile forms, according through the specific *Campylobacter* spp. broth microdilution method (EUCAST, 2020).

Finally, after the biofilm treatment, the media were removed, the wells were washed in 0.9% NaCl solution and the biomass was removed by scraping the wells for 90 s. The cell suspension was seeded on CCDA agar for bacterial count to determine the minimum concentration necessary for inhibition of sessile bacteria in biofilms. ANOVA/Tukey tests were the statistical methods used, determined by R software (p < 0.05).

### Cell Viability Test

VERO cells (monkey kidney fibroblasts) were grown with Dulbecco’s modified eagle medium (DMEM^®^), supplemented with 10% fetal bovine serum, 10 mg/ml streptomycin (Sigma), 100 U/ml penicillin (Sigma^®^), and 40 mg/ml gentamycin, (Sigma^®^) at 37°C in a humidified atmosphere containing 5% CO_2_. VERO cells were seeded at a density of 5 × 10^4^ cells/well in a 96-well tissue culture microplate. After incubation for adhesion, the cells were treated with different concentrations of F6, F14, and F19 and with previously emulsified O6, O14, and O19 oils as controls, at 37°C, 5% CO_2_ for 48 h. The cytotoxicity of the samples was assessed by resazurin assay (Sigma–Aldrich^®^). Then, 10 µl (40 µM final concentration) of resazurin was added per well and incubated for 16–18 h at 37°C in a 5% CO_2_ incubator. The measurements were performed by a spectrophotometer (GloMax^®^) at 594 nm, and the cell viability was expressed as a percentage of viable cells at the end of the experiment (n = 3). Unpaired t-test was the statistical method used to compare significant differences between the formulation and its respective pure oil (p < 0.05).

### Structural Characterization

The structural characterization of F6 formulation and its excipients was assessed by FTIR-ATR, DSC, TEM, and FE-SEM techniques.

In the ATR-FTIR technique, the spectra were recorded with FTIR spectrophotometer with ATR cell (BRUKER IFS 66 v/S or Perkin Elmer SPECTRUM 65) equipment. The spectra were operated in reflectance mode in the range of 4500-500 cm^−1^, with a 2 cm^−1^ resolution. DSC measurements used a cooled TA Q20 calorimeter system. The samples (5 mg) were positioned in aluminum pans and the thermal transitions were assessed in the temperature range from 0 to 100°C, at a heating rate of 10°C/min, under nitrogen flow.

For FE-SEM, the sample was adhered to a stub. After, the stubs were sputtered with gold bath for 120 s at 30 kV. The nanoparticles were visualized in a field JEOL electron scanning microscope (model JSM 5800LV), operating under a variable voltage from 0.3 to 30 kV, with tungsten filament, through the SemAfore 5.21 image capture system software. For TEM, the sample was added to a copper grid. Then, the sample dried with filter paper. The (2% w/w) uranyl acetate was added to provide contrast, and the excess of liquid was removed. Subsequently, deionized water was dropped to the grid, and the excess was removed. The nanoparticles were visualized in a Zeiss–LEO 906 TEM, operating at 60 kV and equipped with an Olympus iTEM CCD camera and image capture software.

## Results

### Screening of Vegetable Oils

A preliminary screening of 28 vegetable oils (**Table 1**) was carried out to select the samples with anti-CJ activity to be used as active molecules in NLC. Among the tested samples, only olibanum essential oil (EO), salvia essential oil (SO), and candeia oil (CO) presented growth inhibition zone (IAL 2383), with diameters around 21–28 mm, in the disc diffusion test (**Table 2**). Then, EO, SO, and CO were used as liquid lipids for the preparation of NLC, entitled as F6, F14, and F19 formulations, respectively. Such systems presented different lipid matrices composition (**Table 3**), being sterically stabilized by Plantaren^®^ and Pluronic 188^®^, used as surfactant and co-surfactant, respectively.

### Long-Term Physicochemical Stability

**Figure 1:**
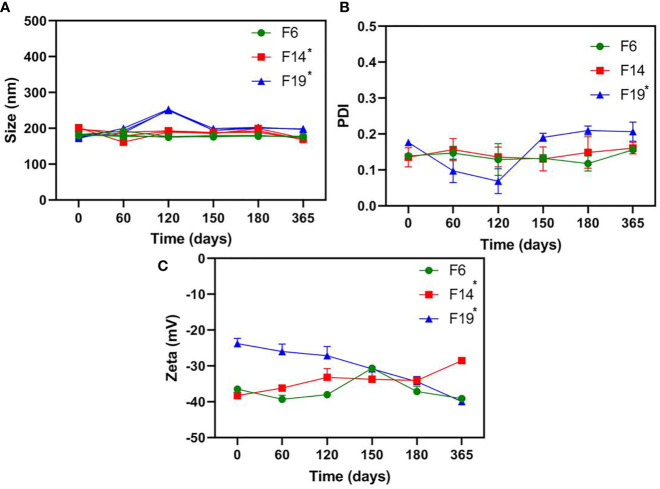
Long**-**term physicochemical stability of NLC formulations, in terms of size **(A)**, PDI **(B)**, and Zeta potential **(C)** values, monitored by DLS for a year (25C°); n = 3. One-way ANOVA plus Tukey *post hoc* tests were used to analyze intragroup statistically significant differences over time; *p < 0.05.

**Figure 1** showed the physicochemical stability results for NLC. The nanoparticle size ranged from 168.8 to 202.6 nm for all the evaluated systems. Among the samples, only F6 did not show any statistically significant change over time. PDI obtained values varied around 0.092–0.220 for all tested NLC, for a year. In this case, only F19 exhibited significant changes (p < 0.05) over time, never surpassing 0.22. Zeta potential values revealed fluctuations from −22.1 to −40.5 mV for all the formulations. F14 and F19 showed statistically significant differences (p < 0.05) in some parameter during the experiment. F6 was the unique NLC that did not exhibit significant differences (p > 0.05) in all the parameters analyzed by the long-term stability study (25°C).

### *In Vitro* Antimicrobial Activity

The formulations F6, F14, and F16 were submitted to the *in vitro* antimicrobial activity tests against CJ strains, through the disc diffusion (free form) and MIC tests for free and sessile forms of CJ (**Table 2**).

In the disc diffusion test, NLC formulations exhibited growth inhibition zones diameters higher (p < 0.05) than its respective controls (**Table 2**). The diameters measured for all the formulations ranged from 35 to 43 mm, while for pure oils the zone of inhibition variation was between 21 and 28 mm.

On the other hand, the determination of MIC in free and sessile forms of CJ (**Table 2**) was not performed for O6, O14, and O19 samples, due to its physicochemical instability, such as: high insolubility to the medium, volatility, and photosensitivity, preventing its analyses. The IAL 2383 CJ (free form) MIC values were 1.56 mg/ml^−1^ for F6 and F14, and 1.25 mg/ml^−1^ for F19 formulation. The MIC results for 10 CJ (free form) were 2.60 mg/ml^−1^ for F6, and 1.56 mg/ml^−1^ for F14 and F19 formulations. Besides, in the MIC of sessile forms (IAL 2383, 468, and 520) determination, F14 and F19 were not able to control the bacterial growth of all the evaluated strains. Only F6 formulation showed ability to inhibit CJ growth in biofilm (sessile form), with MIC values of 0.78 mg.ml^−1^ in all the tested strains (**Table 2**).

### Cell Viability Test

**Figure 2:**
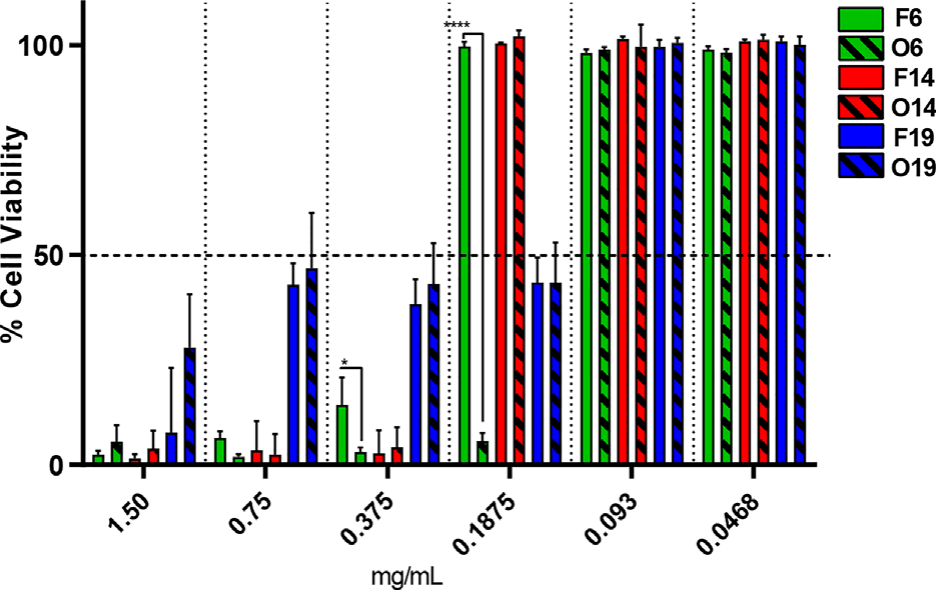
*In vitro* viability test through VERO cell line, after 48 h of treatment for F6, F14, and F19 formulations and its respective control oils (O6, O14, and O19), by resazurin method (n = 3). Significant differences between NLC and its respective control was determined through unpaired t-test; *p = 0.0395 and ****p <0.0001.

The *in vitro* cell viability test was performed through VERO cell line to compare the cytotoxicity of F6, F14, and F19 with its respective oils O6, O14, and O19 (**Figure 2**). The IC_50_ (half maximal inhibitory concentration) values were determined for each sample. In general, it was not observed statistically significant differences between the IC_50_ values of F14 (0.2734 mg/ml) and F19 (0.1466 mg/ml) formulations compared with its respective free oils, O14 (0.3478 mg/ml) and O19 (0.1435 mg/ml), respectively. On the other hand, F6 exhibited IC_50_ value of 0.3529 mg/ml, being statistically significant higher (p < 0.05) than O6 control (0.1424 mg/ml). In addition, F6 exhibited the highest IC_50_ value among all the tested samples.

### Structural Characterization

**Figure 3:**
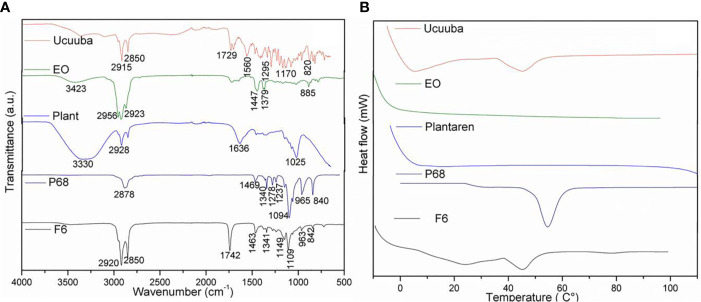
FTIR-ATR **(A)** and DSC **(B)** analyses of NLC (F6) formulation and its excipients. Ucuuba, ucuuba butter; EO, olibanum essential oil; PLANT, Plantaren^®^; P68, Pluronic 188^®^.

**Figure 4:**
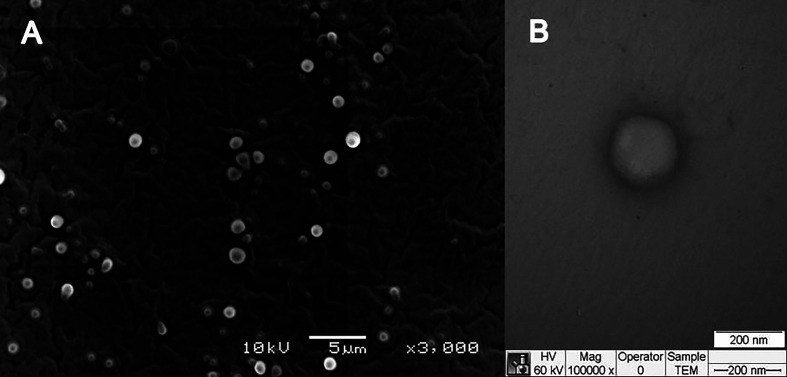
Morphological features of NLC (F6) formulation in terms of FE-SEM **(A)** and TEM **(B)** images.

The structural characterization was performed for F6 formulation and its excipients. In FTIR-ATR analysis, it can be appreciated bands at the regions between 2850 and 2956 cm^−1^ (νO-CH_2_ and νCH), 1729–1742 cm^−1^ (νC = O), and 1109–1170 cm^−1^ (νC = O) in F6, olibanum essential oil (EO), and ucuuba butter spectra (**Figure 3A**), respectively. Plantaren intense band at 3330 cm^−1^ (νO-H) was not evident in F6 spectrum. DSC analysis showed the thermodynamic transitions of excipients and F6 formulation (**Figure 3B**). Ucuuba butter, P68, and F6 showed endothermic peaks related to its melting points, with peaks centered at 45, 56, and 43°C, respectively (Ribeiro et al., 2016; Pardauil et al., 2017). EO and Plantaren did not exhibit any thermal transition during the analysis, due to its liquid state at room temperature. The morphological features of F6 were elucidated by TEM and FE-SEM analyses (**Figure 4**). In both techniques, the nanoparticles showed typical spherical shape (**Figure 4A**) with visible contour, as expected (Ribeiro et al., 2017). The estimating particle size from the micrography (150 nm) (**Figure 4B**) was compatible with that measured by DLS (230 nm).

## Discussion

The screening of 28 bioactive oils to be used as liquid lipids in NLC was performed by the disc diffusion test. Among the tested samples, EO, SO, and CO samples exhibited some zone of inhibition against CJ (**Table 2**), and, therefore, were selected as NLC excipients. The choice of ucuuba and shea butters as solid lipids of NLC was based on previously published works, which demonstrated its ability to encapsulate hydrophobic molecules (Ribeiro et al., 2017; Castro et al., 2019). In NLC preparation, the ratio between the solid and liquid lipids was based on previous lipids miscibility tests (data not shown), resulting in F6, F14, and F19 formulations with different lipids compositions and concentrations (**Table 3**).

The long-term stability study is an essential test to ensure the shelf-life of novel formulations (Barbosa et al., 2018). Then, the nanoparticle size (nm), PDI (particle size distribution), and Zeta potential (mV) values were followed over a year at 25°C (Rodrigues da Silva et al., 2017). Among the prepared NLC, F6 was the unique stable system (p > 0.05) in all the parameters analyzed (**Figure 1**). In fact, the physicochemical stability of nanostructured systems has been correlated with their successful biological activity (Ribeiro et al., 2018), as observed here. It is worth mentioning, despite the statistically significant changes over time for F14 and F19, all the samples exhibited excellent nanoparticle size (<250 nm), monodisperse size distribution (PDI < 0.2), and highly negative Zeta potential values (>−25 mV) at the end of the experiments, as required for NLC development (Souto et al., 2004).

The antimicrobial activity from the formulations were ensured through different *in vitro* experiments. The disc diffusion test showed that all the formulations exhibited higher zone-of-inhibition diameter than free oils. Moreover, MIC values of CJ in free strains was determined for each formulation (**Table 2**). Regarding the activity anti-CJ sessile strains, F6 was the only sample that was able to inhibit the CJ growth in biofilm. In general, Gram-negative are less sensitive than Gram-positive bacteria to essential oils (Kivrak et al., 2009; Yu et al., 2011). However, it has been also showed that the antimicrobial activity of essential oils against Gram-negative bacteria, such as CJ (Šikić Pogačar et al., 2016), can be a versatile alternative to be used as control and treatment (Vasireddy et al., 2018). It is already known that essential oils can interact with bacterial membrane and cell wall, which modifies its structure, contributing to the release of microbial cell content and microorganism death (Carbone et al., 2018; Di Stefano et al., 2020).

In fact, EO, also known as Frankincense, is a natural multiterpene complex (*Boswellia carterii*) from Somalia that has been applied as antimicrobial agent with success, exhibiting MIC values around 4.0–16.0, 1.5–8.3, 4.0–12.0, and 2.0–12.8 mg.ml^−1^ for *Staphylococcus aureus*, *Bacillus cereus*, *Escherichia coli*, and *Proteus vulgaris*, respectively (Van Vuuren et al., 2010). Despite the EO antimicrobial activity, its loading by NLC was essential to enhance its solubility, organoleptic properties and stability; decrease cytotoxicity, photodegradation and volatility, enabling its further use as a pharmaceutical formulation.

In addition, the biofilm formation is a strategy adopted by CJ to survive in hostile conditions. The pathogens are more resistant when organized into biofilm than into planktonic cells (Reuter et al., 2010). Its presence has many implications in food industry, once it creates a persistent source of contamination (Donlan, 2002), in which the control is still a challenge (Van Houdt and Michiels, 2010). The nanostructured size, physicochemical stability and hydrophobicity of NLC can improve the nanoparticle permeation through the expanded and sponge-based shape lipid barrier of the biofilm matrix (Melo et al., 2017), reaching the bacteria in the sessile form. In here, F6 formulation was efficient in controlling CJ in the sessile form at a concentration of 0.78 mg.ml^−1^, which could be further *in vivo* tested as an alternative to control the free and sessile forms of CJ.

Another essential parameter to be ensured by a DDS is its safety. Thus, through the cell viability test, we observed that F6 was the less cytotoxic formulation, being almost twice less cytotoxic than its respective control (**Figure 2**). In this case, NLC exerted a protective effect against the cytotoxicity of free essential oils (Campos et al., 2018). Regarding to the other formulations, there was no significant difference in cell viability or for IC_50_ values in comparison to the respective pure oils, which means that the improvement of antimicrobial activity from NLC was not followed by an enhancement of oils cytotoxicity, as required.

Therefore, considering the excellent long-term stability, *in vitro* antimicrobial activity and cytotoxicity results, F6 formulation was chosen as the best NLC as anti-CJ agent. Then, its structural characterization was performed to understand its supramolecular arrangement. These data are especially relevant, since the structural organization of natural excipients in NLC is still scarce.

Typical NLC spectroscopy profile was confirmed for F6 by FTIR-ATR (Ribeiro et al., 2017) (**Figure 3A**). In general, F6 spectrum was based on the overlapping of the most of ucuuba butter and EO bands, as expected, once they were the major components of F6. However, there is a strong evidence of surfactant and lipid matrix interaction, probably given by hydrogen bonds interactions, showing a complex F6 molecular organization.

In DSC analysis, the calorimetric transitions of the excipients in NLC demonstrated that F6 preserved the thermal properties of its solid lipid (ucuuba butter) matrix (**Figure 3B**). It was also observed a slightly decrease in its melting point (2°C), due to the EO incorporation, which contributed with the structural disorganization of the solid lipid matrix (Carbone et al., 2018). In addition, there was no evidence of any excipient degradation during DSC analysis, confirming the thermal stability up to 100°C. Finally, the morphology of NLC was provided by FE-SEM (**Figure 4A**) TEM (**Figure 4B**) analyses. In both techniques, despite the differences between the sample preparation method and equipment resolution, it was observed typical spherical nanoparticles with well-delimited contour, being homogeneously distributed, as expected for NLC (Ribeiro et al., 2017). Moreover, the estimated nanoparticle size from micrography corroborated the DLS results, considering the particle shrinkage inherent to the sample drying process (Ribeiro et al., 2018).

These abovementioned results confirmed that NLC composed of ucuuba butter and EO is able to be evaluated as anti-CJ agent in specific *in vivo* efficacy assays.

## Conclusions

The use of natural lipids against CJ strains in free and sessile forms is a versatile approach to control and treat this virulent pathogen, especially those strains having antimicrobial resistance. However, the use of these lipids is limited by their physicochemical instability and toxicity. In this work, different NLC composed of natural lipids were prepared and they were shown to exhibit excellent long-term stability over a year at 25°C. Among all NLC, F6 formulation composed of ucuuba butter and olibanum essential oil, was selected as the system that combined the best anti-CJ activity and safety. Supramolecular organization of F6 was detailed, as shown by FTIR-ATR, DSC, TEM, and FE-SEM analyses, confirming an expected NLC typical structural profile. This promising system will be tested *in vivo* assays.

## Data Availability Statement

The raw data supporting the conclusions of this article will be made available by the authors, without undue reservation.

## Author Contributions

LR is the main author. She had the idea of developing the project and carried out the development of NLC in the laboratory. LR, GM, DR, BF, RM, and LG developed the MIC analysis and disk diffusion test. FM, LR, and BF performed the cell culture tests. LR, EP, and MB carried out the characterization, in the coordination of the development of the NLC. LR, BF, and FM contributed to the statistical analysis and writing of the article, LR, BF, LG contributed to the coordination of the project as a whole. All authors contributed to the article and approved the submitted version.

## Funding

This research was funded by Coordenação de Aperfeiçoamento de Pessoal de Nível Superior–CAPES-(#88887.336865/2019-00) and National Institute of Science and Technology in Theranostics and Nanobiotechnology –INCT-Teranano (CNPq/CAPES/FAPEMIG, Grant # CNPq-465669/2014-0).

## Conflict of Interest

The authors declare that the research was conducted in the absence of any commercial or financial relationships that could be construed as a potential conflict of interest.
